# Lysophosphatidic acid 2 alleviates deep vein thrombosis via protective endothelial barrier function

**DOI:** 10.1515/med-2024-1137

**Published:** 2025-02-06

**Authors:** Ruifeng Bai, Xinyang Yue, Xuan Tian, Huiru Zhao, Ying Liu, Tian Li, Jun Wu

**Affiliations:** Department of Clinical Laboratory, Beijing Jishuitan Hospital, Capital Medical University, Beijing, 100035, China; Department of Clinical Laboratory, Peking University Fourth School of Clinical Medicine, Beijing, 100035, China; Department of Vascular Surgery, Beijing Jishuitan Hospital, Capital Medical University, Beijing, 100035, China; Tianjin Key Laboratory of Acute Abdomen Disease-Associated Organ Injury and ITCWM Repair, Institute of Integrative Medicine of Acute Abdominal Diseases, Tianjin Nankai Hospital, Tianjin Medical University, Tianjin 300100, China; Blood Transfusion Department, Beijing Jishuitan Hospital, Capital Medical University, Beijing, 100035, China; Department of Clinical Laboratory, Beijing Jishuitan Hospital, Capital Medical University, 31 Xinjiekou East St., Beijing, 100035, China

**Keywords:** deep vein thrombosis, lysophosphatidic acid 2, vascular endothelial permeability, endothelial cells, zonula occludens-1

## Abstract

**Background:**

The specific role of lysophosphatidic acid 2 (LPA_2_) in deep vein thrombosis (DVT) remains unclear.

**Methods:**

An inferior vena cava annulus retraction model of DVT was established in wild-type (WT) and global LPA_2_ knockout (*Lpar2* ^*−/−*^) mice. We examined the incidence of DVT, wet weight of thrombus, length of thrombus, assessed endothelial permeability through Evans blue dye assay *in vivo,* cell viability, and endothelial cell (EC) permeability of mouse inferior vena cava ECs *in vitro.* Proteomics, histopathology, immunohistochemistry, and western blotting were employed to investigate the role of LPA_2_ in DVT.

**Results:**

*Lpar2* deficiency increased vascular endothelial permeability and promoted the progression of DVT. Histological examination revealed aggravated inflammation in the thrombus of *Lpar2* ^−/−^ DVT mice. *In vitro*, *Lpar2* ^−/−^ resulted in increased permeability of ECs. Proteomic results indicated that DVT after *Lpar2* ^−/−^ may be related to tight junction (TJ) protein. LPA_2_ agonist, 2-[4-(1,3-dioxo-1*H*,3*H*-benzoisoquinolin-2-yl)butylsulfamoyl] benzoic acid, significantly reduced vascular endothelial permeability as well as increased expression of the vascular endothelial TJ protein zonula occludens-1.

**Conclusion:**

These data provide a novel mechanism of endothelial barrier protection of LPA_2_ in DVT.

## 1 Introduction

Deep vein thrombosis (DVT) was a common complication after orthopedic trauma. Clinical data show that DVT was the third most common cause of vascular death after heart disease and stroke [1], and the incidence of DVT reaches 35% after hip or knee replacement [2], with a mortality of 10% [3]. Effective treatments for DVT included oral anticoagulant drugs such as warfarin and the new representative rivaroxaban, or interventional inferior vena cava filter implantation, whereas 50% of the DVT patients still developed post-thrombotic syndrome [4]. At present, it was recognized that blood flow retardation, blood hypercoagulability, and vascular inner wall injury proposed by Virchow are the three major factors of DVT formation.

Endothelial cell (EC) dysfunction caused by damage to the endovascular wall is a leading cause of DVT [5]. When the endothelial barrier function was damaged, endothelial permeability was enhanced, tissue factor (TF) stored in the lower layer of the endothelium was exposed, and the clotting pathway was activated to induce thrombosis by the von Willebrand factor, platelets, and factor VII [6]. Although it was known that the occurrence and development of DVT were closely related to the formation of endothelial permeability, the mechanism of regulating endothelial permeability and then affecting DVT was not clear. Therefore, an in-depth study of the mechanism by which weakening permeability protects endothelial function is of great significance for the development of new drug targets for DVT.

Lysophosphatidic acid 2 (LPA_2_) was reported to stabilize endothelial barrier function *in vitro*, reduce EC permeability [7,8], and stimulate angiogenesis *in vivo* [9,10,11]. Studies have shown that LPA therapy partially improved lipopolysaccharide-induced epithelial barrier breakdown. Another study also supports the role of LPA in endothelial barrier function. LPA acyltransferase inhibitors can reduce LPA degradation and protect lung leakage *in vivo* [12], enhance the barrier function of corneal ECs, and play a protective role in airway inflammation and airway remodeling [13].

There are six subtypes of LPA receptors, namely LPA_1–6_. Our research group has previously found that the expression of LPA_2_ in the heart after myocardial infarction is increased, mainly from ECs, and the results showed that the cardiac vascular permeability is significantly increased in *Lpar2* ^−/−^ mice. In addition, our study found that the expression of LPA_2_ in pulmonary microvascular ECs was increased after acute septic lung injury, and the pulmonary vascular permeability of *Lpar2* ^−/−^ mice was significantly increased. At the same time, LPA_2_ from carotid vessels increased by 6.2 times after carotid artery ligation [14]. In addition, LPA_2_ had a hundredfold or more affinity with LPA than other LPA receptors [15,16]. In conclusion, it was believed that the upregulated expression of LPA_2_ in ECs under injury conditions had an important regulatory effect on vascular ECs. Therefore, in this study, *Lpar2* ^−/−^ mice were used as the model animal and WT mice as the control group, and endothelial permeability was taken as the focus of DVT research.

## 2 Materials and methods

### 2.1 ECs

The mice (purchased from the Animal Center of Capital Medical University) were sacrificed using carbon dioxide. The whole chest and abdominal cavity were dissected layer by layer through the median incision of the chest and abdomen to reveal the entire chest and abdominal cavity. The artery envelope and surrounding adipose tissue were carefully separated under a 10-fold microscope. It was cut into a 1 mm × 1 mm blood vessel graft and attached to the inner side of the blood vessel to the 6-hole plate. After standing for 5–10 min in a 5% CO_2_ incubator at 37°C, ECM complete culture medium containing 20% FBS was added and continued to be cultured in the incubator [17]. The culture medium was changed every 3 days, and flow cytometry was performed with a CD31 label. All cells used in this experiment ranged from P2 to P6.

### 2.2 Endothelial permeability

The cells of the transwell culture plate (coated with 1% gelatin) were incubated at 37°C for 30 min. The stable growth of EC cells of 2–6 generations was digested with pancreatic enzymes and inoculated in the transwell chamber at a density of 1 × 10^4^ (200 μL), and 1 ml of complete culture medium was added into the lower chamber. The cells were cultured in a 5% CO_2_ incubator at 37°C for 24 h, and the serum of DVT mice was stimulated for 48 h after starvation for 12 h. The cells were washed twice with PBS, and 100 μL of phenol-red free DMEM (containing 1 μg/mL of FITC-d-glucoside) was added to the top chamber of the double-layer chamber, and 600 μL of phenol-red free DMEM was added to the bottom chamber of the double-layer chamber, and cultured in the incubator for 45 min. The fluorescence intensity of FITC-dextran was measured by placing 100 μL of liquid in a 96-well plate with a black transparent bottom.

### 2.3 Animal experiments

Mice were anesthetized by isoflurane. The skin was disinfected, and the inferior vena cava and abdominal aorta below the liver were exposed by microsurgical forceps. Ligation of small branches of the inferior vena cava to prevent the establishment of collateral circulation affects thrombosis. The inferior vena cava was wrapped with a 7-0 suture close to the left renal vein, a 27G needle was placed close to the inferior vena cava, and the needle and inferior vena cava were tied together with a 7-0 suture wrapped around inferior vena cava. After the 27G needle was extracted, the inferior vena cava could restore partial blood flow. Using 5-0 sutures to close the abdomen layer by layer, the mice were safely returned to the cage. Phenotypic assessment was performed at the sampling time point, and blood was taken from the eyeball under anesthesia by intraperitoneal injection of tribromoethanol, and then sampling was performed.

### 2.4 Evans blue dye (EBD)

EBD solution (normal saline, 0.5%) was prepared, 200 μL was injected into the tail vein of each mouse 30 min before sampling, and the mice were killed by neck removal (to reduce the interference of anesthetic drugs on the vascular permeability of mice, the faster the action is, the better), the vena cava was removed, weighed, and added with 500 μL formamide, and the supernatant was centrifugally removed after 48 h of 55°C water bath. Using formamide as blank, the supernatants were measured at 620–740 nm spectrophotometrically.

### 2.5 Hematoxylin and eosin staining

The inferior vena cava was isolated and fixed by immersion in 4% paraformaldehyde, embedded in paraffin, and sectioned at 5 μm thickness. The slices were roasted, dewaxed, hydrated, and then stained with hematoxylin. Then, the slides were separated with 1% hydrochloric acid alcohol, and the cytoplasm and other components were further stained with an eosin staining solution. The excess staining solution was rinsed with running water, and the staining effect was observed under a microscope. The nucleus was blue, and the cytoplasm and other interstitial spaces were red. The slides were further dehydrated and sealed with neutral gum. Pathological changes in the lung tissues were examined under a light microscope.

### 2.6 Immunohistochemistry staining

The slicing preparation process is described above [18]. Inferior vena cava was embedded in paraffin, sectioned (5 mm), and mounted on glass slides. After dewaxing, antigen retrieval was performed using citrate buffer and then permeabilized with 0.1% Triton X-100 for 10 min. After being blocked by hydrogen peroxide, the slides were immunostained with an anti-fibrinogen antibody (1:100, Abcam, ab34269) overnight at 4°C and rewarmed at 37°C for 30 min. Subsequently, sections were incubated with ready-to-use undiluted secondary antibodies conjugated with HRP for 30 min at 37°C. Subsequently, diaminobenzidine staining was used for 5 min at room temperature, and the nuclei were stained with hematoxylin for 2.5 min at room temperature. The stained sections were observed under a light microscope. Images were analyzed using the Image-J software.

### 2.7 Western blotting

The left lung obtained from each group was frozen in liquid nitrogen and stored at −80°C. Tissue samples from various groups were homogenized with protein extraction reagents containing protease inhibitors. For ECs of different groups, the cell samples were rinsed twice with 1× PBS and lysed with the lysis buffer. The treated cells were mechanically scraped off with a rubber scraper and centrifuged at 12,000 *g* for 25 min, and the supernatant was collected. Protein concentrations of both tissues and ECs were determined by using Pierce TM BCA Protein Assay Kit (Thermo Fisher Scientific, USA, 23225). Samples of supernatant containing 40 μg protein were heated to 72°C for 10 min and then separated by sodium dodecyl sulfate-polyacrylamide gel electrophoresis in 10% gels. Protein bands were electroblotted onto polyvinylidene difluoride membrane and blocked with 5% BSA for 1 h. The membranes were incubated with primary rabbit polyclonal antibody anti-VE-cadherin (1:1,000, Abcam, ab205336), anti-zonula occludens-1 (ZO-1; 1:1,000, Abcam, ab216880), and anti-claudin-5 (1:1,000, ThermoFisher, 35-2500). Primary antibodies were diluted in 5% BSA overnight at 4°C before the membranes were incubated with the secondary antibodies. The immunoblots were detected by using SuperSignal West Pico Plus (ThermoFisher Scientific, 34577). The protein band intensity was measured using Image J software.

### 2.8 Statistical analysis

GraphPad Prism 8 and SPSS 21.0 software were used for statistical analysis. The data are expressed as mean ± SD. One-way ANOVA, together with the Tukey test for multiple comparisons after the variance homogeneity test, was used to assess significant differences among groups. Differences were considered statistically significant when *p* < 0.05.


**Ethical statement:** The Animal Care & Use Committee of Jishuitan Hospital approved this study (JL-K2022-016), which was conducted in accordance with the “Regulation to the Care and Use of Experimental Animals (1996)” guidelines of the Beijing Animal Care Council. The authors are accountable for all aspects of the work and for ensuring that questions related to the accuracy or integrity of any part of the work are appropriately investigated and resolved.

## 3 Results

### 3.1 LPA_2_ deficiency causes increased incidence of DVT in mice

**Figure 1:**
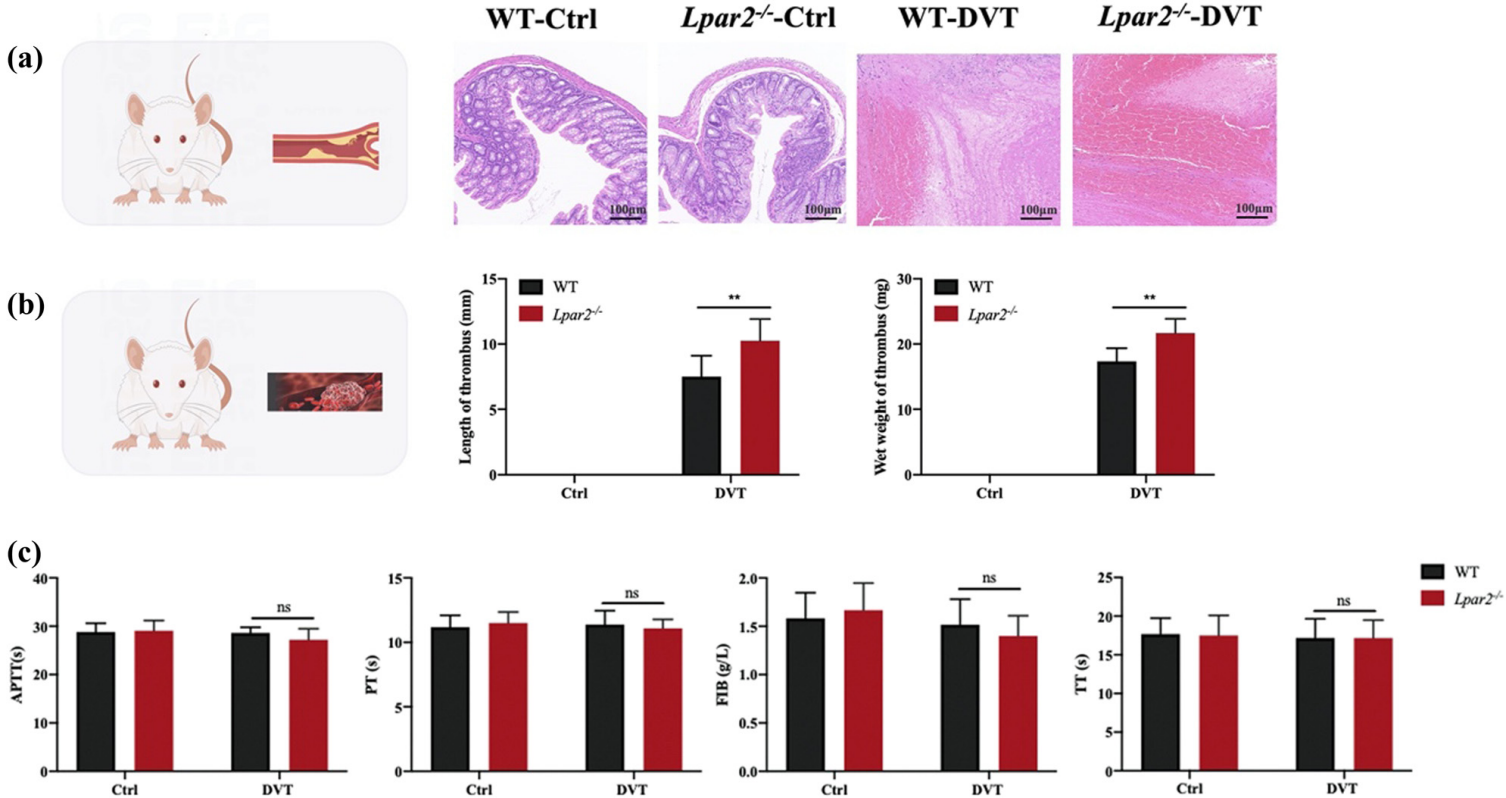
LPA_2_ deletion causes more DVT production in mice. (a) HE staining showed that DVT and inflammatory cell infiltration increased in *Lpar2* ^−/−^ mice. Scale bar = 100 μm. (b) Statistical charts of thrombus length and wet weight in each group. (c) Changes in APTT, PT, FIB, and TT in plasma. Compared with mice in the WT-DVT group, *p < 0.05 and **p < 0.01.

HE staining showed that the vascular ECs in blank and blank groups were intact and orderly, and no inflammatory cell infiltration was observed. Mice in the WT-DVT group and the *Lpar2* ^−/−^-DVT group revealed red thrombus with infiltration of neutrophils, monocytes, macrophages, and other inflammatory cells around the thrombus wall, and inflammatory cells in the *Lpar2* ^−/−^-DVT group were significantly increased (Figure 1a). Compared with the WT-DVT group, the wet weight and length of thrombosis in the *Lpar2* ^*−*/−^-DVT group were significantly increased (Figure 1b). Compared with the WT-DVT group, the APTT, PT, FIB, and TT in the *Lpar2* ^*−*/−^-DVT group were decreased (Figure 1c). It is suggested that LPA_2_ reduced the occurrence of DVT.

### 3.2 Increase of DVT after LPA_2_ deletion may be related to the enhancement of endothelial permeability

**Figure 2:**
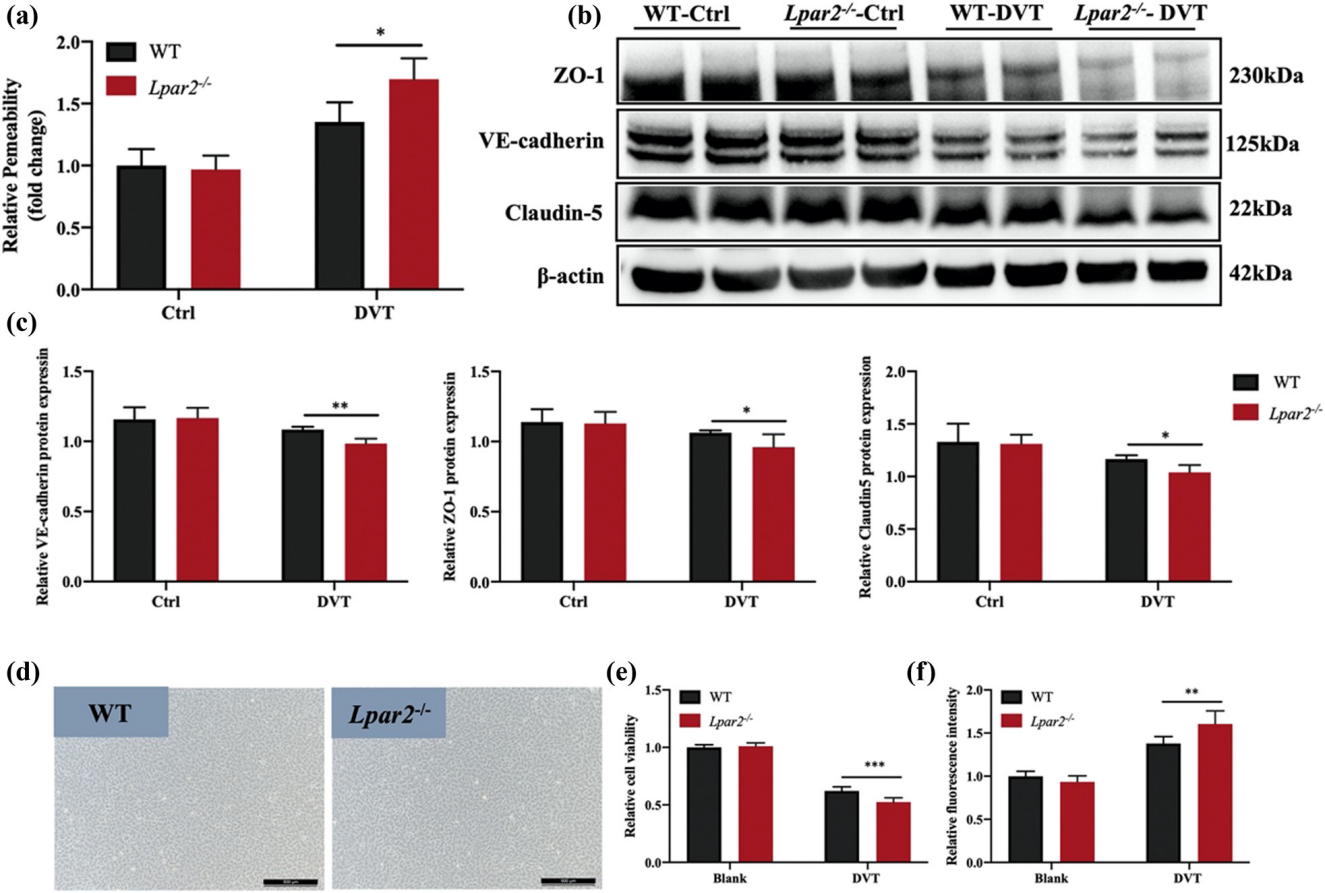
LPA_2_ deletion induced enhanced endothelial permeability of inferior vena cava in mice. (a) Evans Blue assay found that the vascular permeability of inferior vena cava in mice in the *Lpar2* ^−/−^-DVT group was significantly enhanced. (b) The detection of connexin, which reflects endothelial permeability, showed that the expressions of VE-cadherin, ZO-1, and claudin-5 in the inferior vena cava of mice in the *Lpar2* ^−/−^-DVT group were lower than those in the WT-DVT group. (c) Statistical analysis of VE-cadherin, ZO-1, and claudin-5 results. (d) Optical microscopic representation of primary vascular ECs. Scale bar = 50 μm. (e) CCK-8 activity of isolated primary aortic ECs and serum-induced cell damage in mice after DVT. (f) ECs were spread in the transwell chamber, and 70 kDa FITC-dextran was added to detect the permeability of ECs in each group. Compared with mice in the WT-DVT group, *p < 0.05 and **p < 0.01.

Disruption of endothelial function resulted in changes in vascular permeability, and excessive increase in vascular permeability resulted in vascular leakage. TF stored in the subendothelial layer exposes blood and promotes DVT production. Compared with the WT-DVT group, the vascular permeability of the inferior vena cava of mice in the *Lpar2* ^*−*/−^-DVT group was significantly enhanced (Figure 2a). The expressions of VE-cadherin, ZO-1, and claudin-5, which reflect endothelial permeability were decreased in the *Lpar2* ^*−*/−^-DVT group (Figure 2b and C). The primary vascular ECs of WT and *Lpar2* ^*−*/−^ mice were separated, and the results of studies *in vitro* showed that cell activity of *Lpar2* ^*−*/−^ mice ECs decreased more under injury conditions (Figure 2e), suggesting that the loss of Lpar2 may affect the activity and function of ECs, lead to enhanced vascular permeability (Figure 2f), and increase the production of DVT. These results suggested that the loss of Lpar2 disrupts vascular homeostasis and leads to the breakdown of vascular endothelial barrier function, which might be a pivotal reason for the increased formation of DVT in *Lpar2* ^*−*/−^ mice.

### 3.3 LPA_2_ endothelium-specific deletion causes an increased incidence of DVT in mice

**Figure 3:**
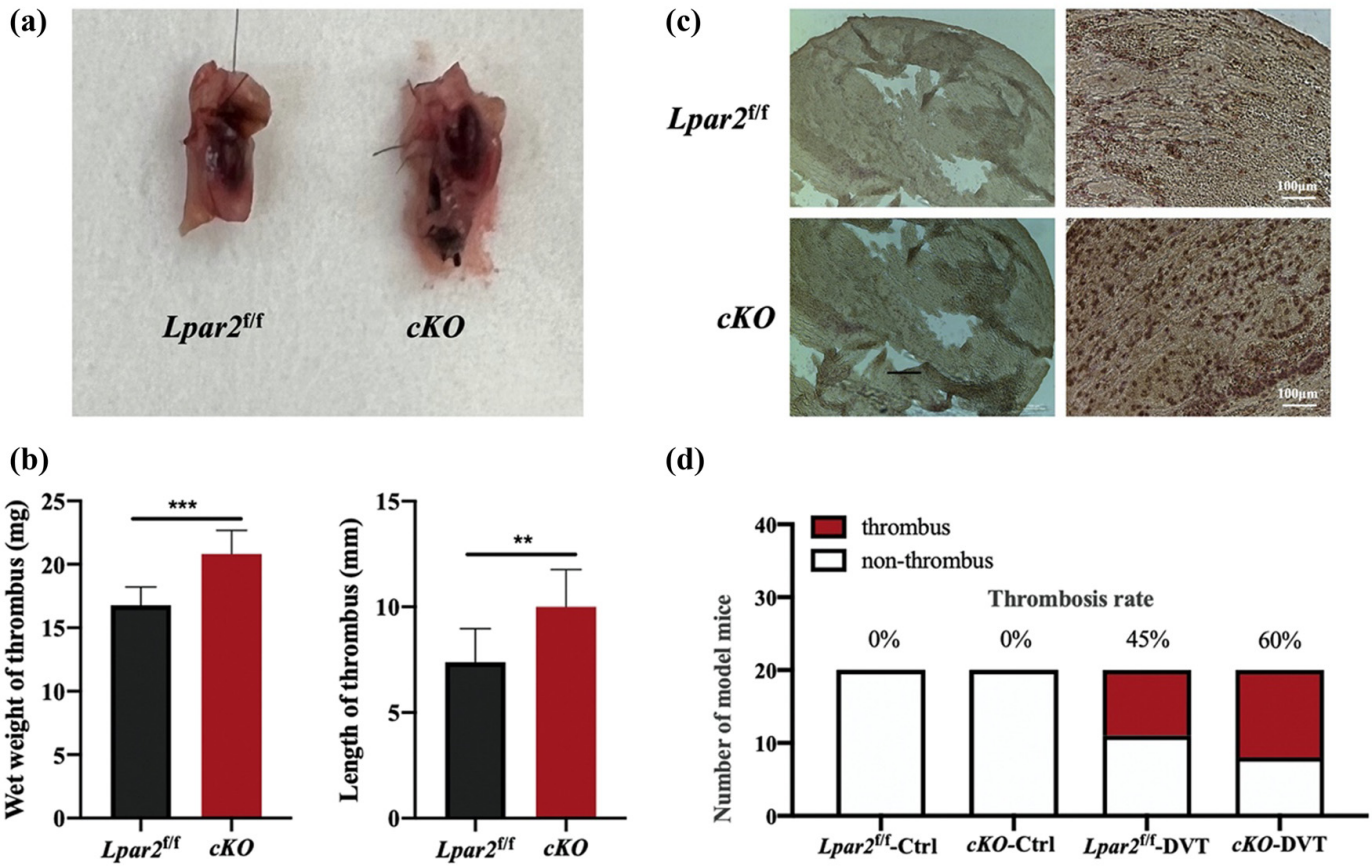
Increased release of inflammatory cytokines from LPA2-deficient ECs. (a) A visual view of thrombus. (b) Immunohistochemical staining of thrombus. Scale bar = 100 μm. (c) Statistical charts of thrombus length and wet weight in each group. (d) Thrombosis rate of mice in each group. Compared with mice in the *Lpar2* ^*f*/*f*^ group, *p < 0.05 and **p < 0.01.

To determine the functional significance of LPA_2_ in DVT, we introduced LPA_2_ endothelium-specific knockout mice. By gross observation, venous thrombosis was formed in both the WT-DVT group and the *Lpar2-cKO*-DVT group. The vascular wall adhesion to the surrounding tissue was light in the *Lpar2* ^*f*/*f*^-DVT group and seriousness in the *Lpar2-cKO*-DVT group (Figure 3a). Fibrinogen promotes platelet aggregation, increases blood viscosity and peripheral resistance, causes EC damage, migrates chemotactic monocytes/macrophages to the intima, and promotes red blood cell adhesion and thrombosis. Immunohistochemical staining results showed that fibrinogen increased in the thrombi of *Lpar2-cKO*-DVT group mice (Figure 3b). Compared with the *Lpar2* ^*f*/*f*^-DVT group, the wet weight and length of the thrombus in the *Lpar2-cKO*-DVT group were significantly increased. Fibrinogen increased in the *Lpar2-cKO*-DVT group (Figure 3c). The thrombus formation rate of *Lpar2* ^*f*/*f*^ mice and *Lpar2-cKO* mice was compared by ligation of the inferior vena cava-induced DVT model. The experimental results showed that the thrombolytic rate of WT was 45%, and that of *Lpar2* ^*−*/−^ mice was 60%. LPA_2_ inhibited the occurrence of DVT (Figure 3d).

### 3.4 Enhanced endothelial permeability of *Lpar2-cKO* mice was associated with tight junction (TJ) proteins

**Figure 4:**
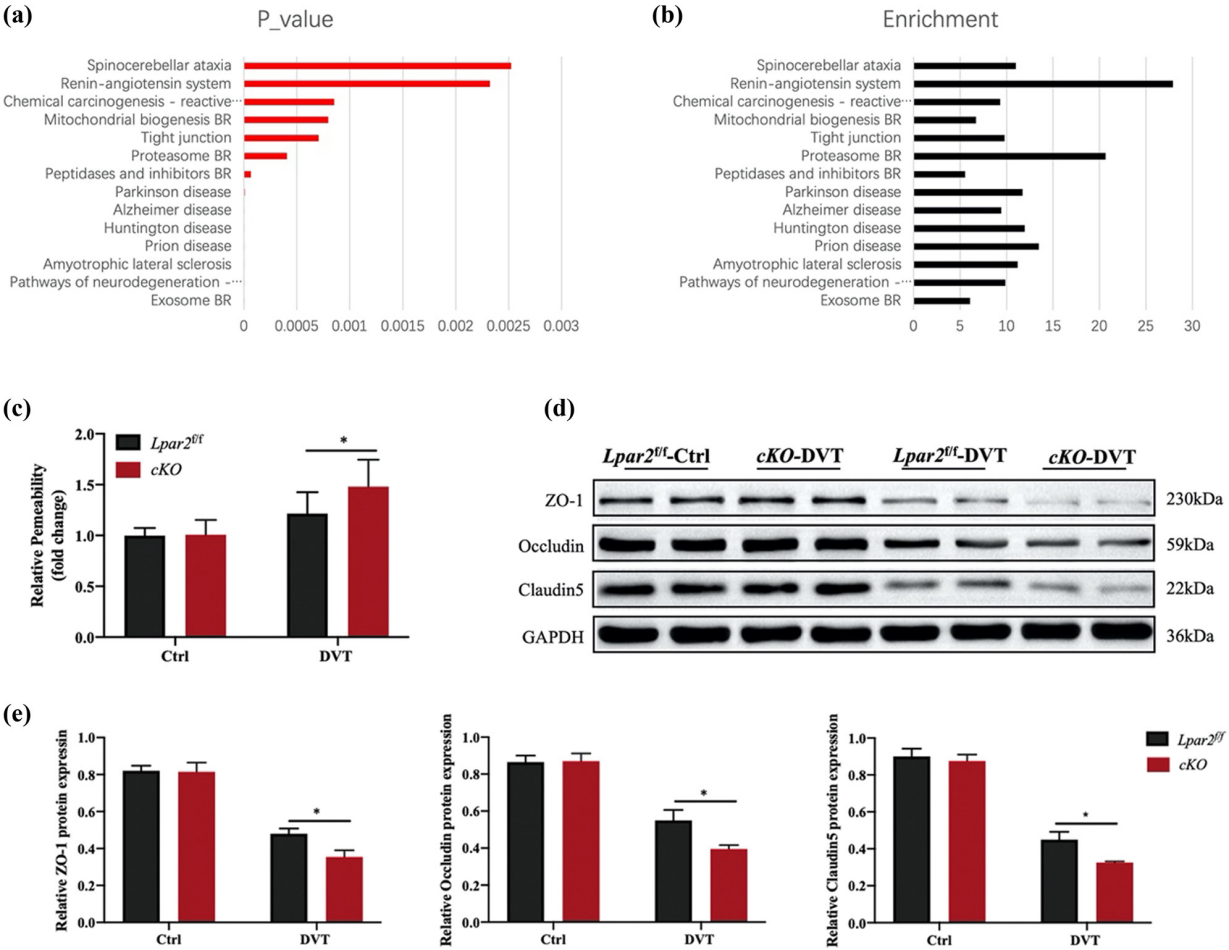
Endothelial permeability upregulated in *Lpar2-cKO* mice. (a) *P* value of the downregulated pathway. (b) Each group down pathway enrichment. (c) Detection of vascular permeability of mice inferior vena cava. (d) Expressions of ZO-1, occludin, and claudin-5 in the inferior vena cava of mice. (e) Statistical analysis of ZO-1, occludin, and claudin-5.Compared with mice in the *Lpar2* ^*f*/*f*^ group, *p < 0.05 and **p < 0.01.

In order to further explore the reasons for increased DVT formation in mice after endothelium-specific knockout of LPA_2_, we performed proteomic analysis on the inferior vena cava of *Lpar2-cKO* and *Lpar2* ^*f*/*f*^ mice. The results showed that the TJ protein pathway was downregulated in *Lpar2-cKO-*DVT mice (Figure 4a and b). For further verification, we tested the vascular permeability of mice inferior vena cava, and the results showed that the endothelial permeability was enhanced in *Lpar2-cKO-*DVT mice, indicating that LPA_2_ indeed plays an important role in endothelial function (Figure 4c–e).

### 3.5 Endothelial permeability was weakened after LPA_2_ drug activation or adenovirus overexpression

**Figure 5:**
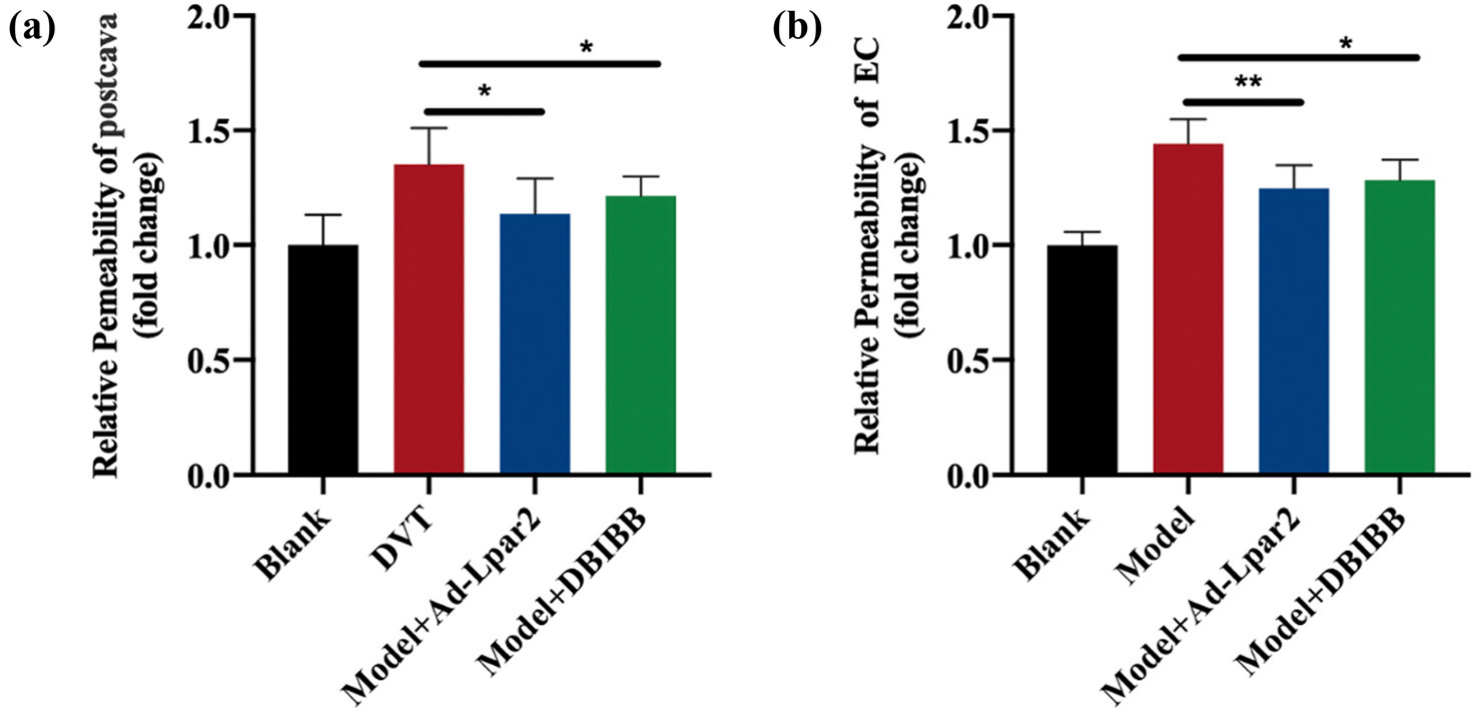
Reduced endothelial damage due to LPA_2_ overexpression. (a) Detection of vascular permeability of mice inferior vena cava. (b) Isolation of primary mouse inferior vena cava vascular ECs to induce permeability detection after cell injury. Compared with mice in the *Lpar2* ^*f*/*f*^ group, *p < 0.05 and **p < 0.01.

The results *in vivo* showed that the vascular permeability of the inferior vena cava was significantly weakened after 2-[4-(1,3-dioxo-1*H*,3*H*-benzoisoquinolin-2-yl)butylsulfamoyl] benzoic acid (DBIBB) activation or LPA_2_ adenovirus overexpression in *Lpar2* ^*f*/*f*^ mice (Figure 5a). The same results were shown in primary ECs extracted *in vitro* after DBIBB activation or LPA_2_ adenovirus overexpression (Figure 5b), suggesting that overexpression of LPA_2_ affected EC function. These results suggested that adenovirus overexpression of DBIBB and LPA_2_ improved endothelial function and effectively maintained endothelial homeostasis, which played an important role in maintaining the vascular barrier and thus reducing the production of DVT.

## 4 Discussion

### 4.1 Main interpretation

**Figure 6:**
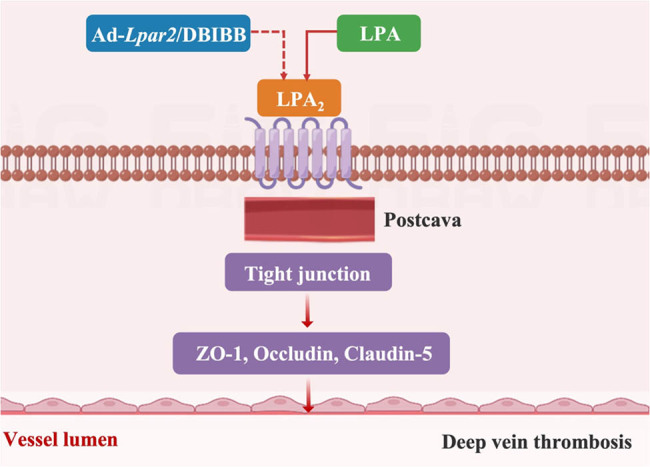
Graphic abstract. LPA2 upregulates the expression of TJ proteins ZO-1, occludin, and claudin-5, maintains endothelial barrier function, and decreases DVT. DVT reduced overexpression of LPA2 by Ad-Lpar2/DBIBB.

Vascular endothelium plays a very important role in thrombotic diseases [19,20]. In this study, we demonstrated that LPA_2_ played a protective role in inferior vena cava annulus retraction-induced DVT, which was associated with endothelial barrier function. *Lpar2* deficiency increased vascular endothelial permeability in the inferior vena cava. *In vitro*, activation of LPA_2_ with DBIBB mitigated DVT by decreased endothelial permeability (Figure 6).

The endothelial barrier function played a critical role in modulating DVT. Previous studies reported a 6.2-fold increase of LPA_2_ in carotid artery LPA_2_ after carotid artery ligation [14,21]. Our group found that LPA_2_ was upregulated in mice after myocardial infarction and that elevated LPA_2_ levels were mainly due to increased LPA_2_ expression in vascular ECs [22]. This attracted our interest because it highlighted the potential role of LPA_2_ in the pathogenesis of DVT. Prior to our findings, no data were available regarding the role of LPA_2_ in DVT. How LPA_2_ was involved in the pathogenesis of DVT is our big concern.

ECs play a pivotal role in the maintenance of vascular homeostasis. An increase in vascular permeability leads to exposure of subcutaneous collagen, activation of platelets and coagulation factor XII, and initiation of the endogenous coagulation system. The injured ECs released TF, activated clotting factor Ⅶ, and activated the exogenous clotting system. *Lpar2* deficiency led to a marked induction in vascular endothelial permeability of the inferior vena cava. By applying the inferior vena cava annulus retraction-induced DVT model in Lpar^2−/−^ mice, we showed that LPA2 was required for vascular endothelial permeability, further confirming the substantial role of LPA2 in endothelial barrier function. Cultured primary ECs from Lpar^2−/−^ mice showed an increase in endothelial permeability characterized by relative fluorescence intensity. These results indicated that Loss of Lpar2 caused an increase in mortality after DVT, possibly due to the destruction of endothelial barrier function, resulting in increased vascular endothelial permeability.

Although it was suggested that LPA played a protective role in endothelial barrier function [7,23,24], it was unclear which LPA receptors helped to mediate the key steps of vascular endothelial permeability in veins. Studies suggested that the knockdown of LPA_1_ increased lung endothelial permeability [25]. LPA via LPAR6 induced actin stress fiber formation and increased brain capillary EC permeability [12,26]. Our previous study showed the potential benefit of LPA_2_ in vascular endothelial homeostasis following cardiac ischemia [22]. These observations provided evidence that different LPA receptors are used for various functions of ECs. In our current research, we demonstrated that the function of LPA_2_ in endothelium permeability by using *Lpar2* ^*−*/−^ mice could stimulate the expression of VE-cadherin, ZO-1, and claudin-5. All these results indicated that LPA-LPA_2_ plays an important protective role in DVT.

DBIBB is an agonist of LPA_2_, and it has been reported that DBIBB can increase intestinal crypt survival and enterocyte proliferation and decrease apoptosis, thus reducing gastrointestinal radiation syndrome [16]. DBIBB could also contribute to vascular endothelium homeostasis and protect cardiac function and myocardial remodeling in mice after ischemic injury [22]. In our study, DBIBB reduced endothelial permeability in WT mice. These results suggested that LPA_2_ is an original regulator for DVT, which was similar to the gastrointestinal radiation syndrome study [16].

## 5 Limitations

The present study has some limitations. First, we focused on the study that LPA2 inhibited DVT by acting in the endothelium and thus inhibited DVT; whether it regulated coagulation is something that we have only preliminarily explored. Second, our study demonstrated that increased DVT after endothelial-specific knockdown of LPA2 was associated with the TJ proteins ZO-1, occludin, and claudin-5, whereas the one that played the most pivotal role is unknown. Finally, the specific pathway by which LPA2 regulates TJ proteins on the endothelium remained unknown. Further studies are needed in the future to better understand the exact mechanisms.

## 6 Conclusion

In conclusion, our data revealed for the first time a novel, selective, and causal role of LPA_2_ activation in mediating endothelial barrier dysfunction in the DVT model *in vivo* and *in vitro*. The beneficial effects of LPA_2_ were mediated by the restoration of endothelial AJ and TJ proteins, VE-cadherin, ZO-1, and claudin-5. The activation of LPA_2_ with DBIBB protected against endothelial barrier function and improved DVT. These findings suggested that targeting LPA_2_ and its downstream signaling pathways may prove to be an innovative therapeutic strategy for the treatment of DVT.
